# Supplementary figures and images for: Infection of humanized mice with a novel phlebovirus presented pathogenic features of severe fever with thrombocytopenia syndrome
Source: PLoS Pathog. 2021 May 11;17(5):e1009587. doi: 10.1371/journal.ppat.1009587 (PMC8139491; doi:10.1371/journal.ppat.1009587)

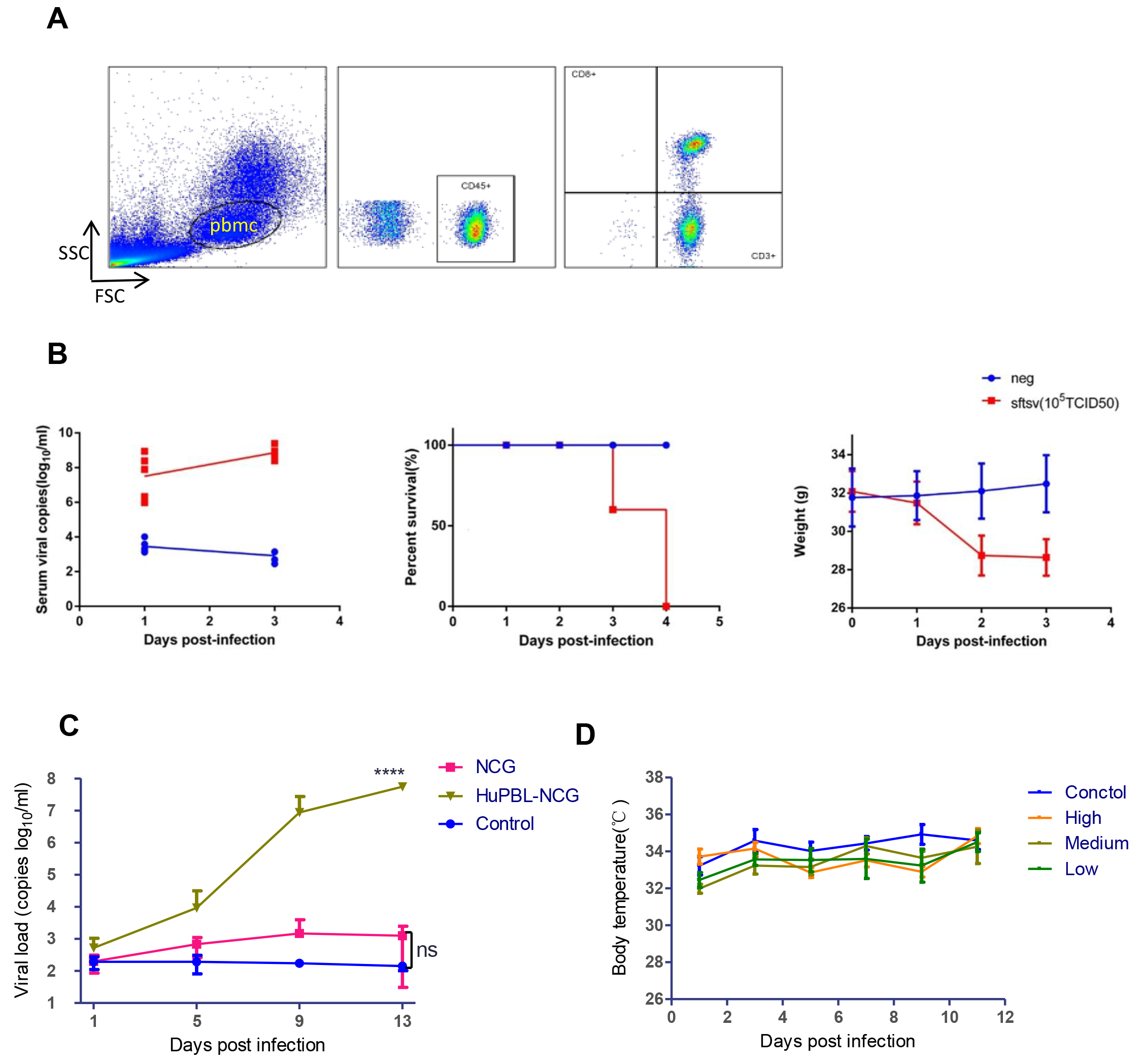

Supplement: S1 Fig — (A) HuPBL-SCID mice flow cytometry analysis of human CD45+, CD3+ and CD8+ cells. (B) Effect of on SFTSV infected AG6 mice. The number of viral RNA copies in blood of SFTSV-infected mice, survival curve and weight change curve. (C) IL-2 knockout in a SCID background NCG mice remain refractory to SFTSV infection, while be susceptible to virus infection after humanized with PBMCs. (D) Body temperature during SFTSV infection of HuPBL-NCG mice. The results presented that no obvious difference was observed among different virus titers infection groups. Data are shown as mean±SEM of three independent experiments.(****p <0.001). (TIF) [file ppat.1009587.s001.tif]

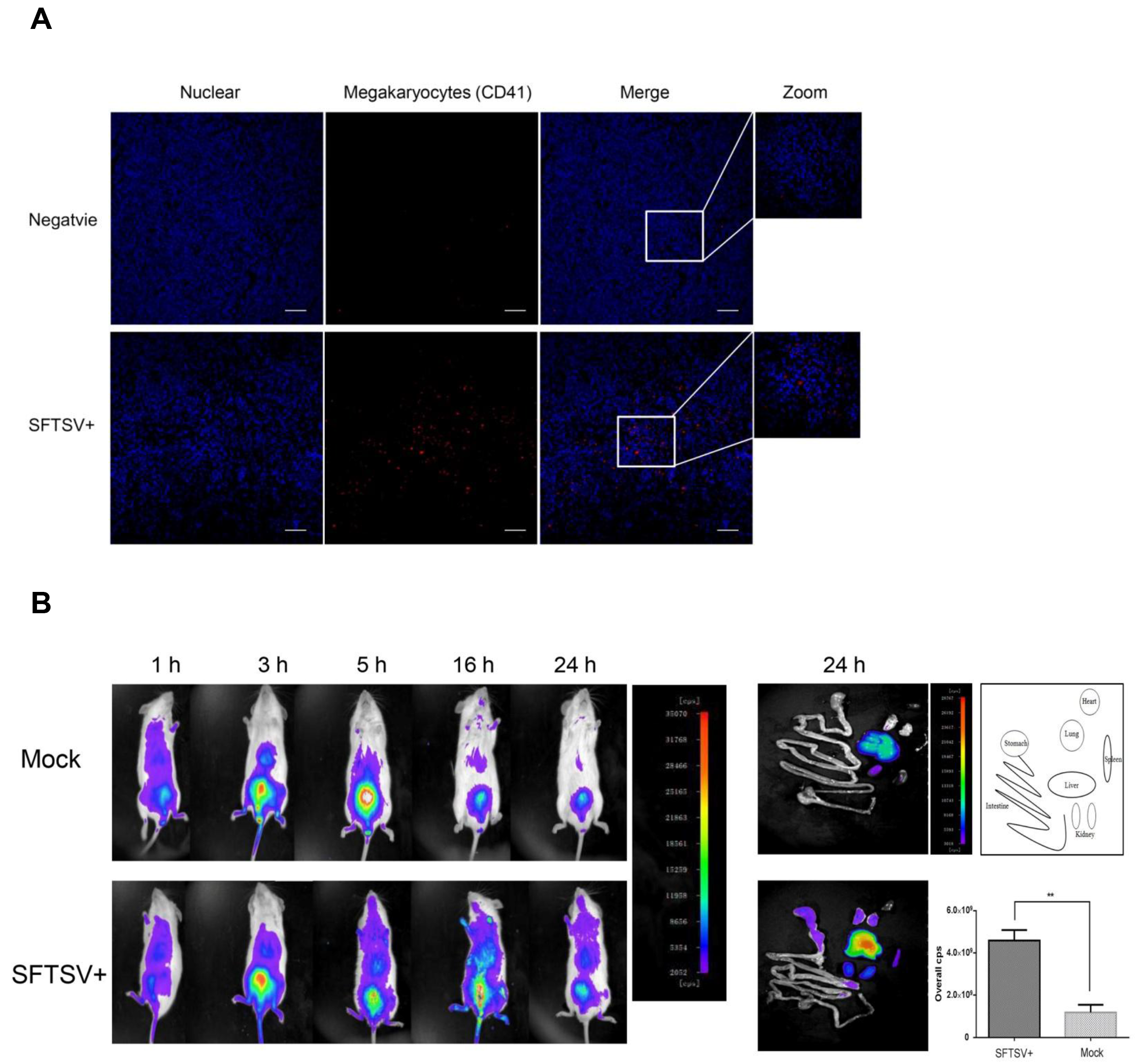

Supplement: S2 Fig — (A) Immunofluorescence assay detected the number of megakaryocytes increased significantly in the spleen, Bar = 100 μm. (B) Fluorescence labled Gn specific antibody detected SFTSV distribution in vivo. After intravenous injection of fluorescence labled Gn specific antibody in HuPBL mice, the Far-infrared fluorescence of whole animal or various organs was acquired at the indicated time points by NightOwl LB 983. Data are shown as mean±SEM of three independent experiments.(**p <0.01). (TIF) [file ppat.1009587.s002.tif]

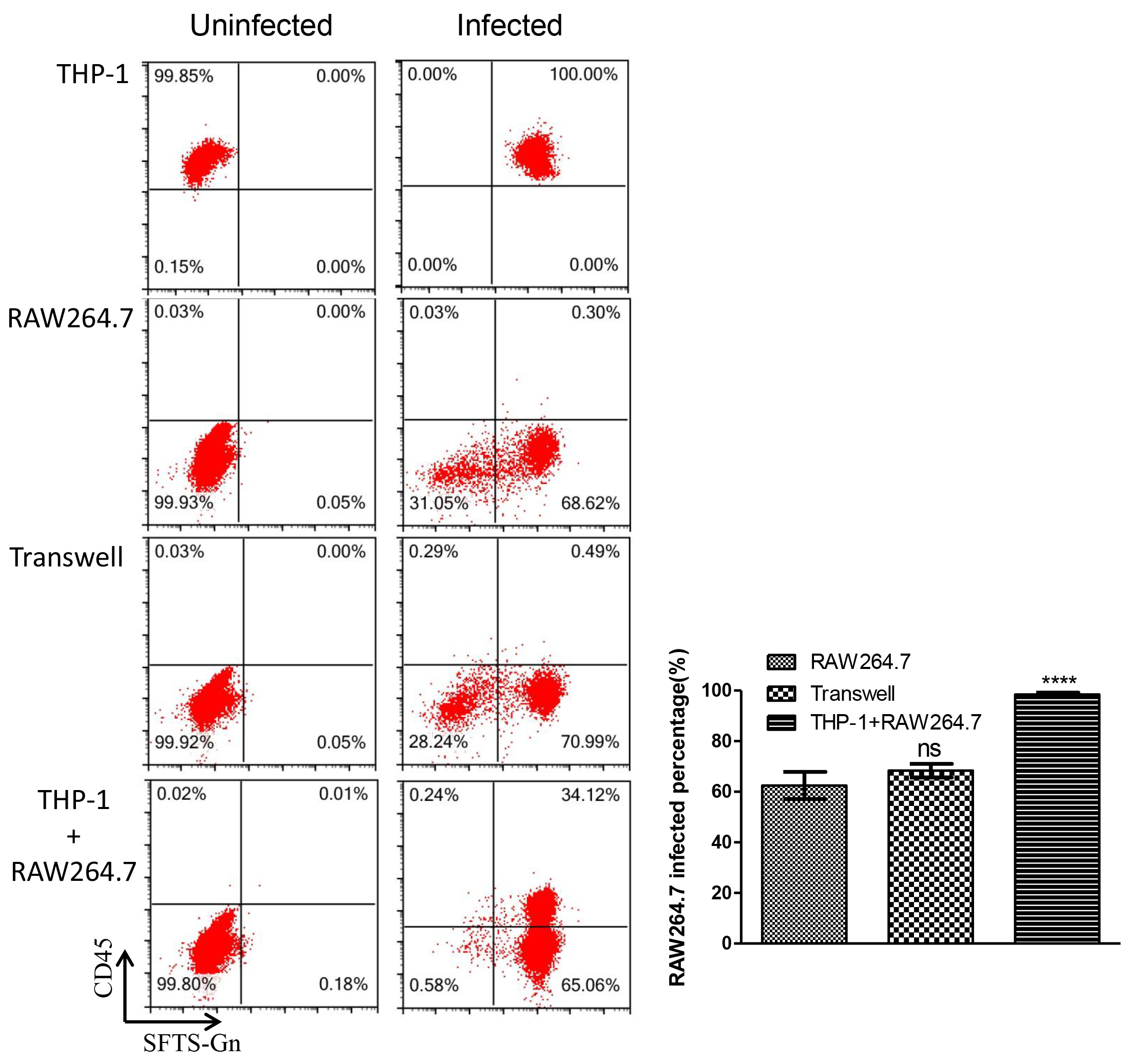

Supplement: S3 Fig — Combining CD45-APC stained THP-1 with Gn-FITC stained SFTSV-infected cells, which demonstrated all THP-1 were infected with SFTSV and THP-1 significantly improve the virus infection of RAW264.7 cells in vitro via direct cell-cell contact. Data are shown as mean±SEM of three independent experiments. (****p <0.001). (TIF) [file ppat.1009587.s003.tif]

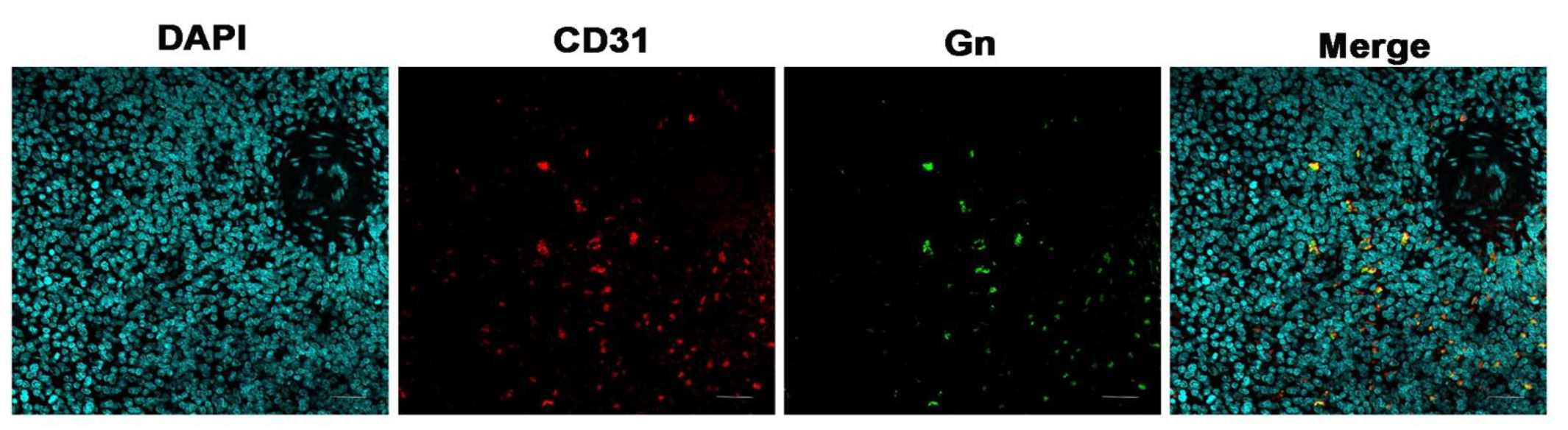

Supplement: S4 Fig — Detection and colocalization of SFTSV antigens in tissues of Rhesus Macaques Spleen. The confocal image shows viral Gn protein (in green) accumulating in anti–platelet cell adhesion molecule 1 (PECAM-1; CD31) vascular endothelium cells (in red), Scar bar, 50 μm. (TIF) [file ppat.1009587.s004.tif]

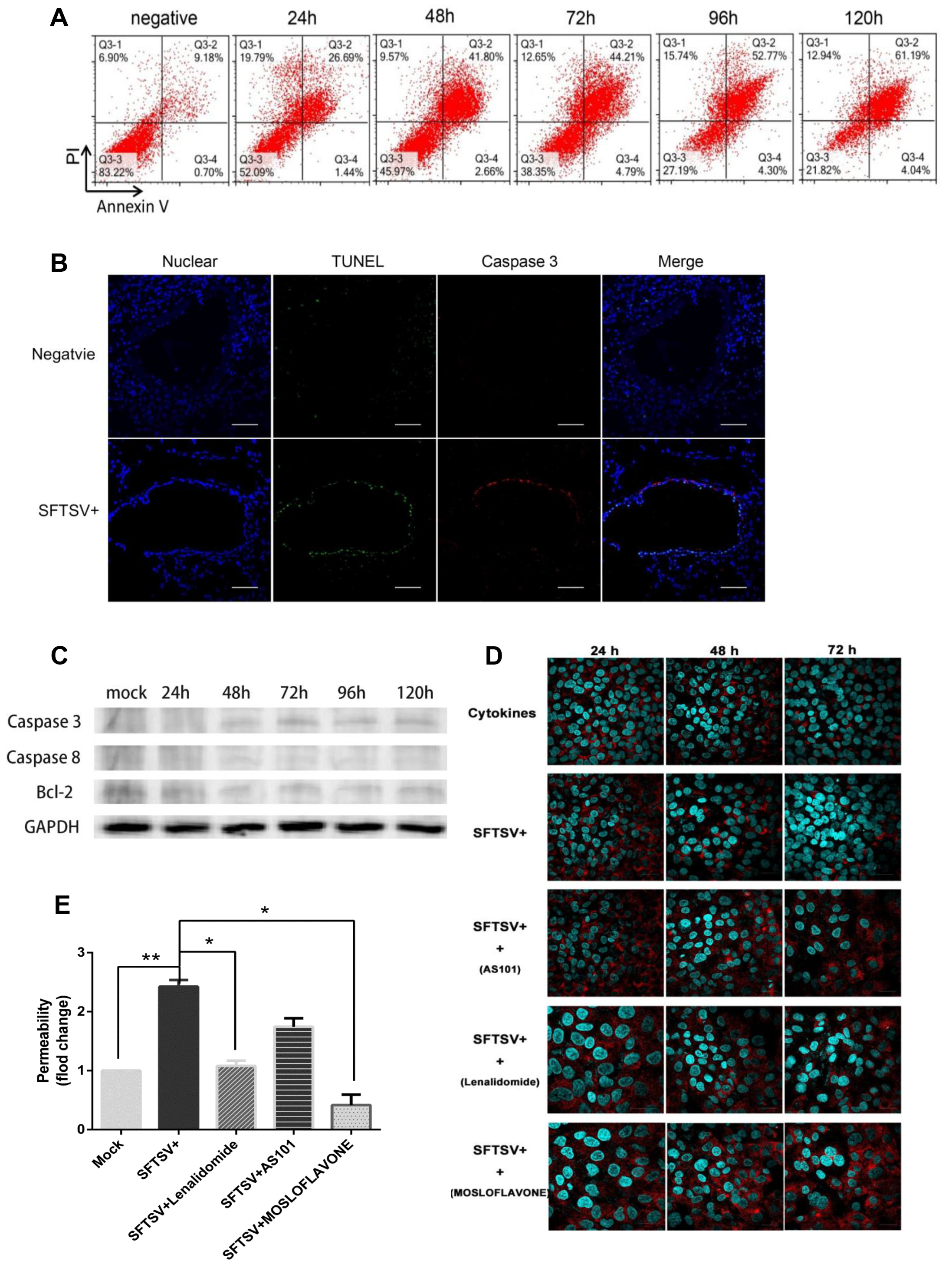

Supplement: S5 Fig — (A) Annexin V-FITC/PI Apoptosis Detection Kit test the apoptosis of virus-infected HUVEC, SFTSV could directly induce apoptosis of endothelial cell. (B) The measurement using TUNEL/Caspase 3 immunofluorescence staining showed that endothelial apoptosis could be observed in virus infected mouse model, Scar bar = 50 μm. (C) Low-dose (MOI = 1) virus infection without cells apoptosis. No significant elevated apoptosis level were observed within 5 days of infection. (D) Dynamic change of three proinflammatory cytokine inhibitors increased the amount of VE-cadherin protein persist on the cell membrane, Bar = 20 μm. (E) Three proinflammatory cytokines inhibitors suppressed SFTSV-directed permeabilizing responses at physiologic concentrations. Data are shown as mean±SEM of three independent experiments. (*p <0.05, **p < 0.01). (TIF) [file ppat.1009587.s005.tif]

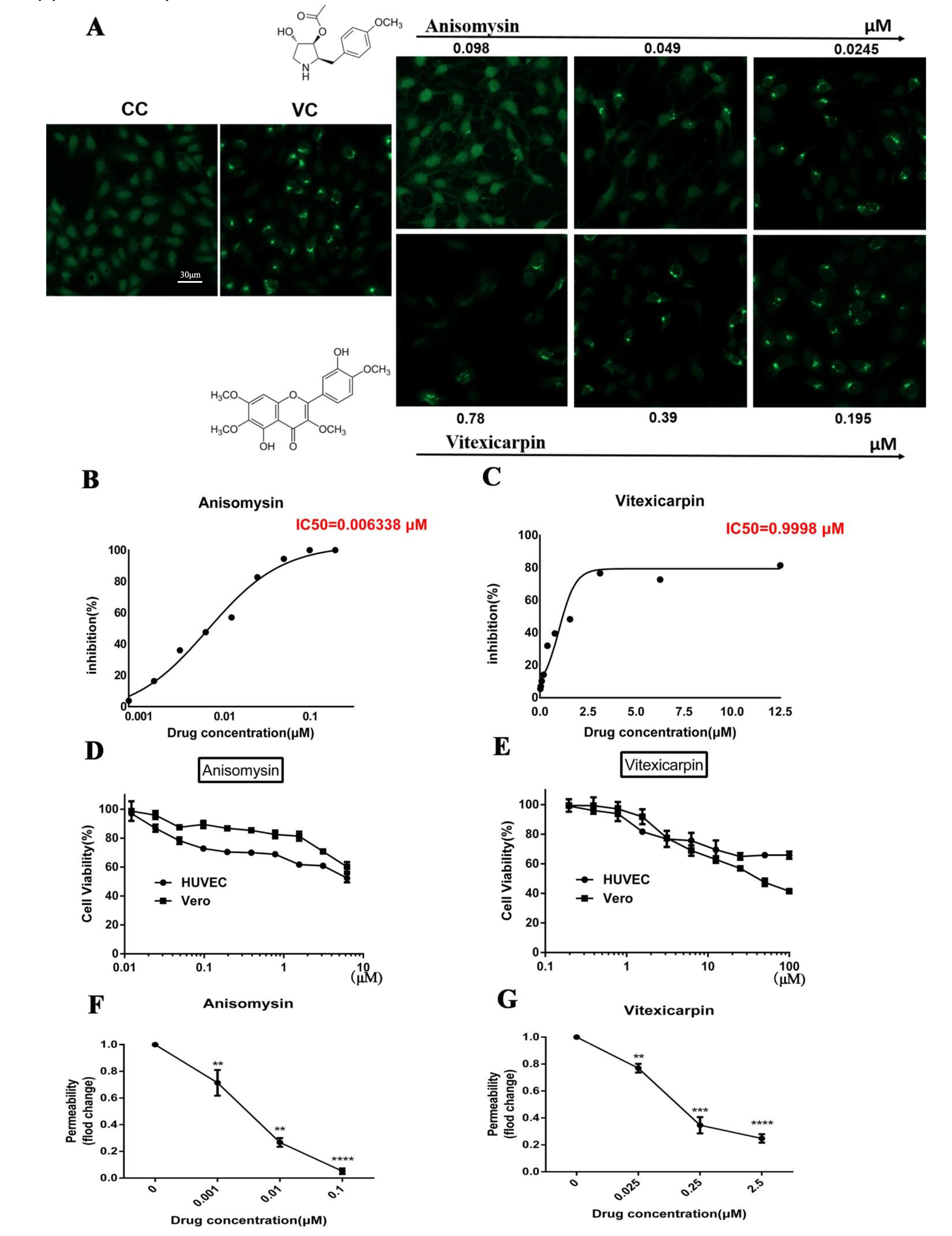

Supplement: S6 Fig — (A-C) The molecular structure of anisomysin and vitexicarpin and the effect of these two drugs on SFTSV, the images are 200x. (D-E) Cytotoxicity of anisomysin and vitexicarpin to HUVEC and Vero cells. HUCEV and Vero cells were incubated with serial concentrations of two drugs and cell viability was measured by CCK-8 kit after 48 h. (F-G) Permeability changes of endothelial cells on HUVECs after anisomysin and vitexicarpin treated. Data are shown as mean±SEM of three independent experiments. (**p < 0.01, ***p <0.005, ****p <0.001). (TIF) [file ppat.1009587.s006.tif]

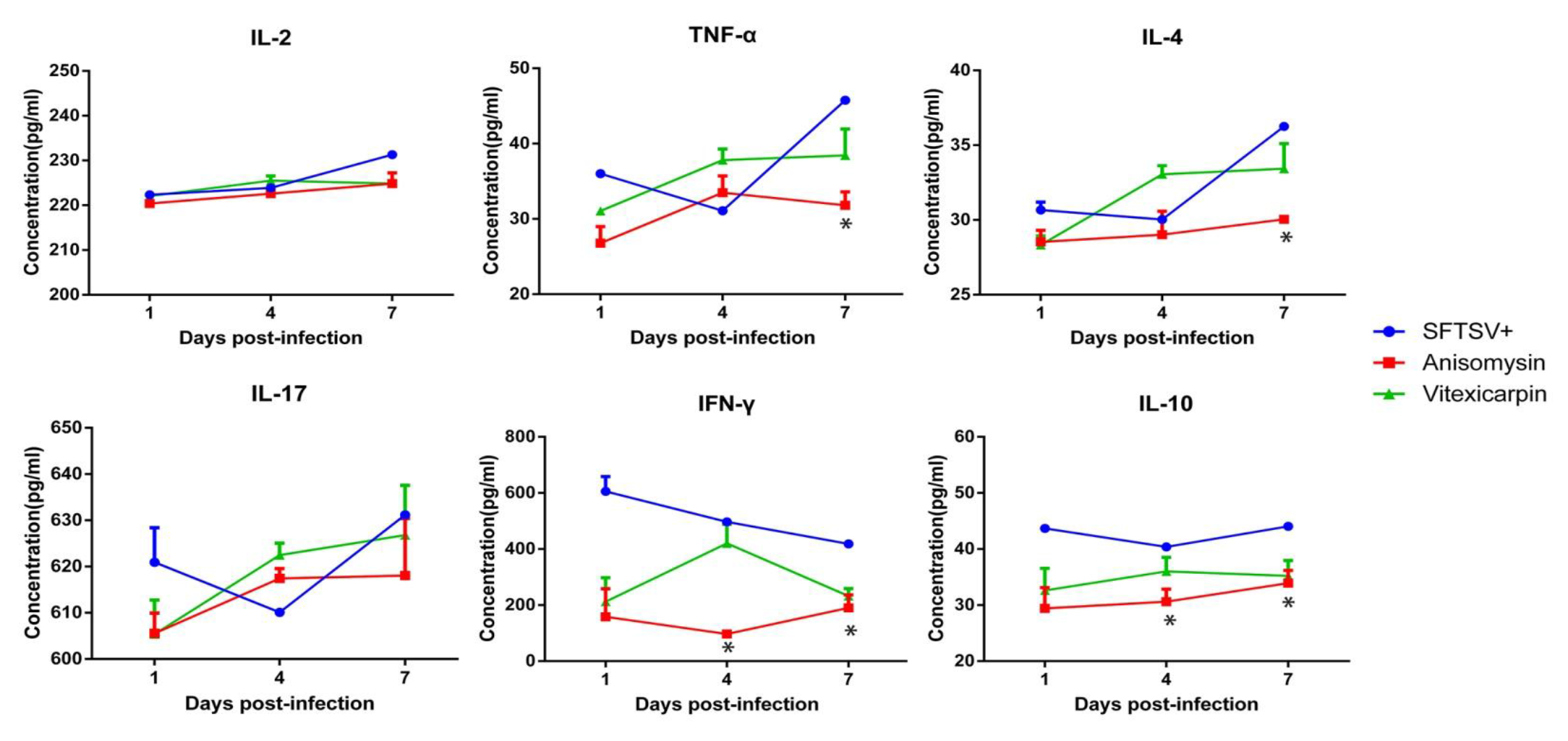

Supplement: S7 Fig — Anisomysin and Vitexicarpin decreased the plasma level of multi-human inflammatory factor. Data are shown as mean±SEM of three independent experiments. *p < 0.05. (TIF) [file ppat.1009587.s007.tif]
