# Supplementary material for: Infection of humanized mice with a novel phlebovirus presented pathogenic features of severe fever with thrombocytopenia syndrome
Source: PLoS Pathog. 2021 May 11;17(5):e1009587. doi: 10.1371/journal.ppat.1009587 (PMC8139491; doi:10.1371/journal.ppat.1009587)
Supplement: Attachment — Submitted filename: Response to Reviews.docx [file ppat.1009587.s008.docx]

**Point-by-point Response**

Dear Dr. wu,

Thank you very much for submitting your manuscript "Infection of humanized mice with a novel phlebovirus presented pathogenic features of sever fever with thrombocytopenia syndrome" for consideration at PLOS Pathogens. As with all papers reviewed by the journal, your manuscript was reviewed by members of the editorial board and by several independent reviewers. In light of the reviews (below this email), we would like to invite the resubmission of a significantly-revised version that takes into account the reviewers' comments.

Reply: We are thankful to the reviewers and the Editorial Board for their tremendous efforts in providing insightful comments and giving us the opportunity for the resubmission of our revised manuscript.

Please also address the following points:

1.) It is important to clearly indicate how NCG mice were reconstituted with PBMC and how inter-donor variation is/can be accounted for in your model. Please also indicate this caveat in your text.

Reply: Human PBMC provided by Jiangsu Provincial Red Cross was isolated from the peripheral blood of healthy donors. According to our previously reported method (*Wu et al., JCI, 2018; Wu et al., JCI insight, 2020*) in the construction of Hu-PBL mice model with following modifications: 2x10^7^ PBMCs from a single donor was injected intraperitoneally into each NCG mouse. Often multiple donors were used in a single experiment but individual mouse always received PBMCs from a single donor and mice were randomly divided for the experiment. Three weeks later, blood was taken from the mice to evaluate the percentage of human CD45. The mice with over 5% human CD45 in PBMC (HuPBL-NCG) was considered as successfully humanized, which were then randomly divided into uninfected control group and infected group. The percentage of successfully humanized rate is mostly over 90% and unsuccessfully humanized mice were discarded. Though the PBMCs isolated from different donors exhibited individual differences, HuPBL-NCG mice receiving human PBMCs from multiple donors were randomly divided into different groups to compensate for the inter-donor variation. We have conducted the *in vivo* animal experiments 4 times, collected PBMCs from almost 30 individuals. Our results showed that the differences in SFTSV replication were not significant in the HuPBL-NCG mice receiving PBMCs from different donors. We have added the description of PBMC reconstitution and inter-donor variation in the discussion section of the revised manuscript.

2.) Beyond permeability, direct measurement of endothelial apoptosis in your mouse model is warranted (ie TUNEL/active caspase strain of infected tissues).

Reply: The reviewer’s comment is well taken. In addition to measure the permeability induced by infection, immunofluorescence and H&E staining were used to evaluate the integrity of vascular endothelium (Fig 5A), which indicated that the continuity and integrity of endothelium markedly decreased as compared with uninfected control mice. For apoptosis, we conducted TUNEL(FITC)/Caspase 3(CY3) immunofluorescence staining and showed that endothelial apoptosis could be observed in virus infected mouse endothelium of the blood vessels as shown below. The TUNEL/ Caspase3 strain of infected tissues results was also added to supplementary figure 5 (S5B Fig).

3.) The text indicates that infection had a suppressive effect on lung endothelial permeability with 3.2X less leakage, but your data indicates the opposite?

Reply: We thank the reviewer for pointing out the erroneous statement. According to the experimental results, infection had a promotive effect on lung endothelial permeability with 3.2-fold increased leakage. We have corrected the error and made a correct statement in the revised manuscript.

Please address. Please provide images with increased magnification for Figure 5. Tissue images with Evans blue and/or albumin/IgG to visbaly demonstrate leakage would strength conclusions.

Reply: The reviewer’s comments and suggestions are well taken.

1. We have replaced Figure 5 with increased magnification as shown below and in the revised manuscript.
2. According to the suggestion, we conducted the Evans blue perfusion test on tissue sections. Compared with the control group, the amount of Evans blue was significantly increased in tissue gaps as shown below.

4.) Minor: please add scale bars to all images, individual data points to all graphs and number of replicates in all legends. “Flod” change should be corrected to Fold change.

Reply: Thanks a lot for your comments. We have corrected them in the revised manuscript.

We cannot make any decision about publication until we have seen the revised manuscript and your response to the reviewers' comments. Your revised manuscript is also likely to be sent to reviewers for further evaluation.

When you are ready to resubmit, please upload the following:

[1] A letter containing a detailed list of your responses to the review comments and a description of the changes you have made in the manuscript. Please note while forming your response, if your article is accepted, you may have the opportunity to make the peer review history publicly available. The record will include editor decision letters (with reviews) and your responses to reviewer comments. If eligible, we will contact you to opt in or out.

[2] Two versions of the revised manuscript: one with either highlights or tracked changes denoting where the text has been changed; the other a clean version (uploaded as the manuscript file).

Important additional instructions are given below your reviewer comments.

Please prepare and submit your revised manuscript within 60 days. If you anticipate any delay, please let us know the expected resubmission date by replying to this email. Please note that revised manuscripts received after the 60-day due date may require evaluation and peer review similar to newly submitted manuscripts.

Thank you again for your submission. We hope that our editorial process has been constructive so far, and we welcome your feedback at any time. Please don't hesitate to contact us if you have any questions or comments.

Sincerely,

Kellie Jurado
Guest Editor
PLOS Pathogens

Sara Cherry
Section Editor
PLOS Pathogens

Kasturi Haldar
Editor-in-Chief
PLOS Pathogens
[orcid.org/0000-0001-5065-158X](http://orcid.org/0000-0001-5065-158X)

Michael Malim
Editor-in-Chief
PLOS Pathogens
[orcid.org/0000-0002-7699-2064](http://orcid.org/0000-0002-7699-2064)

Reviewer's Responses to Questions

**Part I – Summary**

Please use this section to discuss strengths/weaknesses of study, novelty/significance, general execution and scholarship.

Reviewer #1: In this manuscript , authors innovatively developed a SFTSV infection model based on the Hu-PBL-NCG mice that recapitulates many pathological characteristics of SFTSV infection in humans. Authors further revealed that SFTSV infection increased the vascular permeability of endothelial cells by promoting tyrosine phosphorylation and internalization of the adhesion molecule vascular endothelial (VE)–cadherin. In addition, authors found that both virus infection and pathogeninduced exuberant cytokine release dramatically contributed to the vascular endothelial injury. However, the following comments and questions need to be addressed.

Reply:

We thank the reviewer for giving positive and encouraging comments on our work and several insightful suggestions for improvement. We have carefully studied these comments and suggestions and amended the relevant parts in the manuscript. The questions raised by the reviewer are addressed below in detail.

Reviewer #2: The authors reconstituted SCID NCG mice with human PBMC (hPBMC-NCG mice) and infected the mice with severe fever with thrombocytopenia syndrome virus (SFTSV). The authors determined whether the infected mice recapitulated SFTS and could be used as a model system to evaluate antiviral drugs against SFTSV.

Rationale for using hPBMC-NCG mice as SFTSV infection model has not been explained well in this manuscript.

Reply:

We are grateful to the reviewer for reading the manuscript and providing insightful comments. To address the reviewer’s concerns, we provided point-by-point answers to the reviewer’s questions as follows and revised our manuscript accordingly.

Rationale for using the hPBMC-NCG mice:

SFTSV infection has been studied in various animal models, including mice, hamsters and ferrets with various successes. SFTSV-infected C57/BL6 mice were reported with symptoms of thrombocytopenia and leukocytopenia. However, viral replication could not be observed in blood. Even in spleen, viral replication could only be observed 3 days post infection (Jin, Liang et al. 2012). Another murine model, alpha/beta interferon receptor knockout (IFNAR^-/-^) mice, was used to study the pathogenesis of SFTSV and the testing of potential therapeutic and preventive approaches. However, the course of SFTSV infection is short and the infected mice die within 3 to 4 days with high viral load, which makes the pathogenesis distinct from the human infection and allows only a very narrow window for testing drugs. In addition, SFTSV infected reticular cells in the IFNAR^-/-^ mice instead of monocytes/macrophages in human. No thrombocytopenia with a progressive decline in platelets and white blood cells were reported in the SFTSV infected IFNAR^-/-^ mice (Liu, Wu et al. 2014). SFTSV-infected aged ferrets (4 years of age) demonstrated severe thrombocytopenia, reduced white blood cell counts, high fever with high mortality rate. However, ferrets are outbred animals unlike inbred mice and, especially, aged ferrets are quite limited (Park, Kim et al. 2019). In addition, ferret model will not be able to address potential ADCC, ADCP or antibody-dependent enhancement (ADE) effect of SNB02 that carries a human Fc. ADCC, ADCP and ADE are widely observed in antibodies or vaccines against virus infection (see below).

Hu-PBL mice have been widely applied to the study of human virus infection, like HIV, dengue virus, Epstein-Barr virus, Influenza, HBV, Ebola virus, Hantavirus and so on (*Walsh, Kenney et al. 2017*). Hantavirus, a member of Bunyavirales as SFTSV, causes increased vascular permeability and loss of platelets. Humanized mice were successfully infected with Hantavirus displaying high copy numbers of viral genomes in sera and other organs, particularly the lungs. Pulmonary inflammation and drop in human platelet counts quickly followed. Actually, IFNAR^-/-^ mice had been infected with dengue virus, but these models did not provide optimal tools for studies of dengue pathogenesis. Instead, humanized mice were considered a better model for DENV *(**Lai and Chen 2018*). Our HuPBL-NCG mouse model showed that SFTSV infected the monocytes/macrophages and the animals presented thrombocytopenia with a progressive decline in platelets and other multiple pathological features that recapitulate those in human infection. In fact we initially developed this model for evaluating SFTSV neutralizing antibodies and successfully used the model to demonstrate the efficacy of a VHH nanobody which has since been in the clinic development as a therapeutic drug (*Wu, et al., JCI Insight 2020;5(13): e136855*).

**Part II – Major Issues: Key Experiments Required for Acceptance**

Please use this section to detail the key new experiments or modifications of existing experiments that should be absolutely required to validate study conclusions.

Generally, there should be no more than 3 such required experiments or major modifications for a "Major Revision" recommendation. If more than 3 experiments are necessary to validate the study conclusions, then you are encouraged to recommend "Reject".

Reviewer #1: (No Response)

Reviewer #2: 1. The authors cited studies reporting immune incompetent mice supporting SFTSV. NCG is immune incompetent mouse.

1) What did the authors hypothesize to observe by infecting NCG mice with SFTSV, based on the characteristics of NCG mice?

Reply:

Immunodeficient NOD.Cg-PrkdcscidIl2rg^em26^/Nju (NCG) mice were similar as NSG mice, which lacked the IL2 receptor gene in a SCID background, resulting in the absence of murine T, B cells and very small amounts of NK cells. HuPBL-NSG differs from the alpha/beta interferon receptor knockout (IFNAR^-/-^) mice, the model currently used for SFTSV. The course of SFTSV infection of IFNAR^-/-^ mice is short and the infected mice die within 3 to 4 days with high viral load, making its pathogenesis distinct from that in human and not suitable for evaluating anti-viral drugs and antibodies. Given that the HuPBL-NCG mouse model has the reconstituted human immune systems, we hypothesize that HuPBL-NCG mice would support SFTSV replication, respond to the infection with a robust cytokine response and result in virus-related pathogenesis. Another important feature of this HuPBL-NCG mice is that they would give us sufficient window of time for experiment which is not available in the current models.

2) Why did the authors expect to see enhancement of SFTSV infection in hPBMC-NCG mice whose immune systems had been reconstituted with human immune cells, but not in NCG mice?

Reply:

Although NCG mice are IL-2 knockout in a SCID background, the animals remain refractory to SFTSV infection as the added result shown below and supplementary figure 1C. We think that the human PBMCs provide initial targets for the virus to replicate and the virus may spread to murine targets via cell-cell mediated transmission which is more efficient.

****

2. There is no mention of how human PBMC was secured for the study.

Reply:

Human PBMC was prescreened and provided by Jiangsu Provincial Red Cross Organization.

3. The infection model depends on the role of human PBMCs in NCG mice. Although the model looks feasible as a proof of concept, the system might work variably according to the human donor of PBMC, since severity of SFTS appears variable judging from seroprevalence studies. Without consistency guaranteed, could it be called a model system?

Reply:

We thank the reviewer for raising an important question. Though the PBMCs isolated from different donors do have individual differences, we conducted stringent quality control for the infection study after engraftment. Three weeks after human PBMCs (HuPBL) injected intraperitoneally into NCG mice, blood samples from HuPBL-NCG mice were subjected to flow cytometry to determine the percentage of human CD45+, CD3+, CD4+ and CD8+. If the proportion of human CD45+ cells reached more than 5%, HuPBL-NCG mice were selected for the animal model study. We have so far tested PBMCs from dozens of different donors and found that the results have been largely consistent. In rare occasions, the virus failed to replicate and these experiments would be terminated.

4. The reconstituted mice worked for the authors to observe recapitulation of SFTS. But, would it work next time? It is not clear if each hPBMC-NCG mouse was reconstituted with PBMCs from each different person or all hPBMC-NCG mice used were reconstituted with PBMCs from a same person. If the latter was the case, reproducibility of the system might need to be tested using PBMCs from another person.

Reply:

Human PBMC provided by Jiangsu Provincial Red Cross was isolated from the peripheral blood of healthy donors. According to our previously reported method (*Wu et al., JCI, 2018; Wu et al., JCI insight, 2020*) in the construction of Hu-PBL mice model with following modifications: 2x10^7^ PBMCs from a single donor was injected intraperitoneally into each NCG mouse. Often multiple donors were used in a single experiment but individual mouse always received PBMCs from a single donor and mice were randomly divided for the experiment. Three weeks later, blood was taken from the mice to evaluate the percentage of human CD45. The mice with over 5% human CD45 in PBMC (HuPBL-NCG) was considered as successfully humanized, which were then randomly divided into uninfected control group and infected group. The percentage of successfully humanized rate is mostly over 90% and unsuccessfully humanized mice were discarded. Though the PBMCs isolated from different donors exhibited individual differences, HuPBL-NCG mice receiving human PBMCs from multiple donors were randomly divided into different groups to compensate for the inter-donor variation. We have conducted the *in vivo* animal experiments 4 times, collected PBMCs from almost 30 individuals. Our results showed that the differences in SFTSV replication were not significant in the HuPBL-NCG mice receiving PBMCs from different donors. We have added the description of PBMC reconstitution and inter-donor variation in the discussion section of the revised manuscript.

5. Why did the authors not use murine endothelial cells instead of HUVEC for mechanistic characterization of the observed leakage (by Evans Blue) and influences of cytokines of Fig 5 and Fig 6?

Reply:

The primary reason for selecting HUVEC instead of murine endothelial cells to study the mechanism was that SFTSV is a human pathogen and causes pathology in human endothelium. In addition, the virus does not infect murine target cells and it is not clear if it is relevant to conduct mechanistic study on a murine endothelial cell line.

**Part III – Minor Issues: Editorial and Data Presentation Modifications**
Please use this section for editorial suggestions as well as relatively minor modifications of existing data that would enhance clarity.

Reviewer #1:

1. Authors developed SFTSV infection HuPBL-NCG mice model, it is necessary to detect body temperature and blood pressure during SFTSV infection.

Reply:

The reviewer’s suggestion is well taken and we measured the body temperature during SFTSV infection of HuPBL-NCG mice. The results were presented as following. We did not observe temperature elevations in SFTSV infected HuPBL-NCG mice. We speculate that this may be attributable to the fact that different mammals have different regulatory mechanisms of body temperature (*Chan et al., 2018, Neuron*). However, we did not perform the blood pressure measurement since we did not have such an equipment and related experience. In addition, there appears no report in clinic that patients are suffering from blood pressure variation.

2. Considering the influence of transplanted human PBMC, it is better to have a negative control (uninfected) group during SFTSV infection in Fig. 1FG. And what about Platelet counts and WBC counts from low dose virus infected HuPBL-NCG mice during infection? Can this low dose virus infected mice eliminate virus and detect blood cell recovery?

Reply:

We appreciate the reviewer for the insightful comments. Actually, we had a control that was not infected in Fig. 1B (the dotted line). The platelet and WBC counts for the negative control were obtained from this group of mice and normal ranges of platelet and WBC counts in uninfected mice are 480–700 × 10^3^ per µl and 9.5–14.2 × 10^3^ per µl as the gray area indicated in Figure 1FG, respectively.

To address the reviewers’ suggestion on the low dose challenge, we carried out the experiment with HuPBL-NCG mice challenged with low dose (10^3^ TCID_50_) of SFTSV. The virus titer and blood cells were determined. The data was presented as follow.

3. Authors found decreased cellularity and obvious lymphocyte depletion in the red pulp (RP) and the number of megakaryocytes increased significantly in the spleen by immunohistochemistry. It would be helpful if authors could detected by immunofluorescence or flow cytometry at the same time.

Reply: The reviewer’s suggestions are well taken and we carried out the analysis accordingly. The data indicated that the number of megakaryocytes increased significantly in the spleen among SFTSV infected mice as detected by immunofluorescence, consistent with the observations made by immunohistochemistry. The results as shown below were also included in manuscript supplementary figure 2A.

4. Fig. 3A flow chart needs to add the information of horizontal and vertical axes. Fluorescence and corresponding epitope should be marked on the Fig. 3E.

Reply: We have carried out the modifications accordingly.

5. Transwell culture system can effectively prevent direct cell contact. So it would be fine if authors change Fig. 3CD cytokine+SFTSV group by Transwell THP1+SFTSV group. Authors found THP-1 significantly improve the virus infection ability among mouse RAW264.7 cells *in vitro*. It is advisable that to verify it in vivo, such as adding NCG mouse infection group to Fig. 3E.

Reply: The reviewer’s suggestions are well taken and we have completed it accordingly.

In Fig. 3 C&D, we attempted to show that THP-1 facilitated the virus infection of mouse RAW264.7 cells via cell-cell contact but not inflammatory cytokines secretion. To do so, we added proinflammatory cytokines (TNF-a, IL-1β) in the culture medium of RAW264.7 cells and determined if the presence of the cytokines enhanced the infection of the cells.

Per reviewer’s suggestion, we carried out experiments *in vivo* and found that the presence of THP-1 accelerated the expansion of the virus on early days during infection of the mice; however, that did not significantly increase the viral titers in the peripheral blood compared with the animals without THP-1 but challenged with the same viral inoculum (medium) as shown below. The results suggest that the presence of THP-1 in the animals mainly facilitated the early replication of the virus.

6. In this manuscript, could SFTSV directly induce apoptosis of endothelial cell? Do they have similar effects on other endothelial cells?

Reply:

Yes, SFTSV could directly induce apoptosis of endothelial cell as shown by Western blot analysis in figure 6A in the original manuscript. To further substantiate the observation, we carried out additional experiment analyzing virus-infected HUVEC using Annexin V-FITC/PI Apoptosis Detection Kit to determine the apoptosis and found that SFTSV infection could induce apoptosis in HUVEC and the apoptosis increased as the infection proceeded as shown below. Since our focus was on the relationship between the virus infection and vascular permeability, we did not test if virus had similar effects on other endothelial cells.

**Reviewer #2:**

Fig 1: switching positions of C and D is recommended.

Reply: The reviewer’s suggestion is well taken and we have done it accordingly.

Fig 3D: About RAW + THP-1, it cannot be said that THP-1 enhanced SFTSV infection in RAW, since there was no control for THP-1 alone; the data should be reinterpreted.

Reply: The reviewer’s point is well taken. In our previous experiments, THP-1 cells were almost always 100% infected by SFTSV (*Zhang et al., Frontiers in Immunology, 2019*). To address the reviewer’s concern, we performed additional analysis by flow cytometry on infected THP-1 double-stained with anti-CD45-APC and anti-Gn-FITC, and showed that THP-1 cells were 100% infected as shown below which was included in supplementary figure 3 in the revised manuscript. It was demonstrated that almost all RAW264.7 cells were infected in THP-1-RAW264.7 co-culture.

Fig 5B: It seems that description in lines 238 – 244 appears opposite to Fig 5B

Thanks for pointing the mis-statement and we have corrected it.

Line 29; While previous models -> Previous models

We have corrected it.

Line 56; with respect of cells apoptosis -> with respect to apoptosis

We have corrected it.

Line 146; may -> may be

We have corrected it.

Line 177; widely -> wide

We have corrected it.

PLOS authors have the option to publish the peer review history of their article. If published, this will include your full peer review and any attached files.

If you choose “no”, your identity will remain anonymous but your review may still be made public.

**Do you want your identity to be public for this peer review?** For information about this choice, including consent withdrawal, please see our [Privacy Policy](https://www.plos.org/privacy-policy).

Reviewer #1: No

Reviewer #2: No

Figure Files:

While revising your submission, please upload your figure files to the Preflight Analysis and Conversion Engine (PACE) digital diagnostic tool, [https://pacev2.apexcovantage.com](https://pacev2.apexcovantage.com/). PACE helps ensure that figures meet PLOS requirements. To use PACE, you must first register as a user. Then, login and navigate to the UPLOAD tab, where you will find detailed instructions on how to use the tool. If you encounter any issues or have any questions when using PACE, please email us at [figures@plos.org](mailto:figures@plos.org).

Data Requirements:

Please note that, as a condition of publication, PLOS' data policy requires that you make available all data used to draw the conclusions outlined in your manuscript. Data must be deposited in an appropriate repository, included within the body of the manuscript, or uploaded as supporting information. This includes all numerical values that were used to generate graphs, histograms etc.. For an example see here on PLOS Biology: <http://www.plosbiology.org/article/info%3Adoi%2F10.1371%2Fjournal.pbio.1001908#s5>.

Reproducibility:

To enhance the reproducibility of your results, PLOS recommends that you deposit laboratory protocols in protocols.io, where a protocol can be assigned its own identifier (DOI) such that it can be cited independently in the future. For instructions see [http://journals.plos.org/plospathogens/s/submission-guidelines#loc-materials-and-methods](http://journals.plos.org/plospathogens/s/submission-guidelines)
